# Disparities in the use of ambulatory surgical centers: a cross sectional study

**DOI:** 10.1186/1472-6963-9-121

**Published:** 2009-07-21

**Authors:** Seth A Strope, Aruna Sarma, Zaojun Ye, John T Wei, Brent K Hollenbeck

**Affiliations:** 1 Department of Urology, Division of Health Services Research, University of Michigan Health System, Ann Arbor, MI, USA

## Abstract

**Background:**

Ambulatory surgical centers (ASCs) provide outpatient surgical services more efficiently than hospital outpatient departments, benefiting patients through lower co-payments and other expenses. We studied the influence of socioeconomic status and race on use of ASCs.

**Methods:**

From the 2005 State Ambulatory Surgery Database for Florida, a cohort of discharges for urologic, ophthalmologic, gastrointestinal, and orthopedic procedures was created. Socioeconomic status was established at the zip code level. Logistic regression models were fit to assess associations between socioeconomic status and ASC use.

**Results:**

Compared to the lowest group, patients of higher socioeconomic status were more likely to have procedures performed in ASCs (OR 1.07 CI 1.05, 1.09). Overall, the middle socioeconomic status group was the most likely group to use the ASC (OR 1.23, CI 1.21 to 1.25). For whites and blacks, higher status is associated with increased ASC use, but for Hispanics this relationship was reversed (OR 0.84 CI 0.78, 0.91).

**Conclusion:**

Patients of lower socioeconomic status treated with outpatient surgery are significantly less likely to have their procedures in ASCs, suggesting that less resourced patients are encountering higher cost burdens for care. Thus, the most economically vulnerable group is unnecessarily subject to higher charges for surgery.

## Background

In an effort to lower medical costs and improve the efficiency of health care,[1] increasing numbers of surgeries are being performed on an outpatient basis.[2] Little data exists to guide the decision as to where a procedure should be performed.[3,4] For this reason, the choice of surgical setting is often relegated to patient or provider preference. For many patients, personal spending on health care is a significant concern.[5] Many patients have significant co-payments,[6] and for the 45 million uninsured,[7] the total cost of health care drives treatment decisions. The push towards high deductible health plans will further place the cost of care on patients.[8] Thus, patients increasingly find their personal expenditures to be a significant factor in health care decision making.

Decreasing out of pocket payments is especially important for patients of lower socioeconomic status because the high cost of medical care falls disproportionately on them. Though lower income families spend less out of pocket on health care compared to high income families, the burden of these payments is considerably higher.[9] In 2003, one third of individuals living below federal poverty standards spent more than 10% of their income on health related expenses.[8] These expenditures have serious consequences, with half of personal bankruptcies related to medical expenses.[10]

Decreasing personal expenditures for medical care can be achieved through the use of ambulatory surgical centers (ASCs). ASCs provide a lower cost alternative to hospitals for the provision of surgical services. [11-13] Medicare increased this cost advantage for more procedures under new reimbursement guidelines instituted in January 2008.[11] Since many insurance companies develop guidelines similar to Medicare reimbursements for their own policies,[14] and encourage their patients to use these environments,[15] patients with co-payments or co-insurance experience substantial reductions in their out of pocket expenses with greater use of ASCs for outpatient surgery.[3,16]

As lower cost alternatives to hospitals, ASCs provide an advantageous environment for patients of lower socioeconomic status to receive outpatient surgery. However, disparities in the use of ASCs, if evident, are likely to have downstream consequences, including higher expenditures for patients with less ability to afford their care. For this reason, we evaluated the extent to which the use of ASCs varied according to socioeconomic status and race/ethnicity.

## Methods

Using the Agency for Healthcare Research and Quality's State Ambulatory Surgery Database for Florida, all ambulatory surgery procedures at both hospital based facilities and ASCs were obtained for 2005. The dataset provides patient level discharge data for 100% of the ambulatory patients from facilities in state.[17]

### Measuring Socioeconomic Status

The primary exposure of interest was socioeconomic status measured at the level of patient zip codes. Zip code level demographic information was obtained from the US census bureau. Using the method of Diez-Roux,[18] a summary measure of the socioeconomic status at the zip code level was created. Briefly, six constructs were used to represent the socioeconomic status of each small area, including median household income in 1999, the value of owner occupied housing, percent of households with dividend or rental income, the percentage of people who had graduated from high school, the percentage who graduated from college, and the percentage of residents who are employed in managerial, professional, and related occupations. These six components were then standardized into a z-score, and the scores were combined into an overall marker of neighborhood score. The overall scores for zip codes in Florida ranged from -16.19 to 19.43, with higher numbers reflecting more advantaged socioeconomic status. Quintiles of socioeconomic status were then created. Distribution of the components of the neighborhood score is shown in Table 1.

**Table 1:** Socioeconomic status characteristics for all discharges available for study (n = 1,138,127)

	Socioeconomic Status (Quintiles)				
	1 (Lowest)	2	3	4	5 (Highest)

Number of Discharges	208557 (18%)	222873 (20%)	236339 (21%)	233973 (21%)	236385 (21%)

Median SES Summary Score (range)	-3.9 (-16.2 to -1.9)	-0.8 (-1.9 to 0.2)	1.1 (0.2 to 2.2)	3.6 (2.3 to 4.9)	7.5 (5.0 to 19.4)

Wealth/Income					

Median household income ($)	29621	34732	37972	41899	55292

Median value of housing units ($)	73500	87600	97600	123200	170400

Households with interest, dividend, or rental income	23%	32%	38%	44%	53%

Education					

Adult residents who completed ≥ high school	67%	77%	82%	87%	92%

Adult residents who completed ≥ college	16%	22%	27%	35%	48%

Employment					

Employed residents with management, professional, and related occupations	21%	26%	29%	36%	45%

### Study Cohort

We limited our scope of study to procedures commonly performed in both ASCs and hospitals, including lower gastrointestinal endoscopy, upper gastrointestinal endoscopy, cataract surgery, knee arthroscopy, and ambulatory urologic procedures, (Appendix 1). These procedures accounted for 1,138,127 of the 2,662,157 total records (43% of all ambulatory surgeries performed in 2005). Of these discharges, full demographic data was available on 1,122,137 discharges (99% of the possible population). These discharges made up our final study population.

### Statistical Analysis

We first compared the distribution of variables across socioeconomic status cohorts through the use of chi-square tests. Then we assessed our primary outcome of interest, the location of ambulatory surgical procedures: ASC or hospital. We fit logistic regression models using the quintiles of socioeconomic status as the primary exposure of interest. All models were then adjusted for the type of surgical procedure performed, patient insurance status (Medicare or commercial insurance, no insurance or self-pay, and Medicaid or other governmental insurance), age (20 yr ranges), race/ethnicity (white, black, hispanic, other), gender, comorbidity (based on the Elixhauser method),[19] and the level of urbanization of the patient's neighborhood (five category classification of the Urban Influence Codes).[17] SASD classifies race/ethnicity as white, black, hispanic and we grouped other smaller categories together resulting in four mutually exclusive race/ethnhicity groups. We then tested the significance of interactions between socioeconomic status and race/ethnicity through a second set of models that allowed the impact of socioeconomic status to vary by race/ethnicity. These models were run with interaction terms between the race/ethnicity variable and the quintile of socioeconomic status. The significance of the interaction was tested through the type 3 Wald test of the interaction coefficients.

All analysis was performed with SAS Version 9.1.2 (SAS Institute, Cary, NC) using two-sided tests. The probability of Type 1 error was set at 0.05. This study, dealing with publicly available data was exempt from institutional review board approval in accordance with the Code of Federal Regulations, Title 45, Section 46.101.

## Results

Socioeconomic quintiles were balanced with the number of discharges ranging from 208,557 for the lowest group to 236,385 for the highest group (Table 1). The age and racial structures of the cohorts varied significantly with the lowest socioeconomic status groups being younger and more ethnically diverse than the higher groups (Table 2). Medicaid and other governmental insurance was more prevalent in the lowest socioeconomic status group compared to higher groups (11% in lowest versus 3% in highest; p < 0.001). Use of ASCs was lowest for the lowest socioeconomic group (60%) versus the other groups (64% to 67% p < 0.001).

**Table 2:** Patient characteristics by socioeconomic status quintile * All p-values based on Wald chi-square tests.

	Socioeconomic Status (Quintiles)					p-value*
	1 (Lowest)	2	3	4	5 (Highest)	

Number of Discharges	208557 (18%)	222873 (20%)	236339 (21%)	233973 (21%)	236385 (21%)	

No. at ASC	124183 (60%)	143567 (64%)	157743 (67%)	151954 (65%)	152636 (65%)	<0.001

Procedure						<0.001

Cataract Surgery	49360 (24%)	52094 (23%)	55089 (23%)	53681 (23%)	49351 (21%)	

Urologic	26881 (13%)	27327 (12%)	29075 (12%)	28349 (12%)	27520 (12%)	

Upper GI	35659 (17%)	35800 (16%)	37711 (16%)	35453 (15%)	32941 (14%)	

Lower GI	87010 (42%)	96355 (43%)	102946 (44%)	104226 (45%)	113070 (48%)	

Knee Arthroscopy	9647 5%)	11297 (5%)	11518 (5%)	12264 (5%)	13503 (6%)	

Age						<0.001

≤ 19	7750 (4%)	6442 (3%)	5659 (2%)	5489 (2%)	6064 (3%)	

20–39	16006 (8%)	16211 (7%)	15474 (7%)	15600 (7%)	14902 (6%)	

40–59	62732 (30%)	64420 (29%)	66793 (28%)	67199 (29%)	75172 (32%)	

60–79	100871 (48%)	108634 (49%)	117674 (50%)	113312 (48%)	110414 (47%)	

80+	21198 (10%)	27166 (12%)	30739 (13%)	32373 (14%)	29833 (13%)	

Race/Ethnicity						<0.001

White	130718 (63%)	174813 (79%)	197407 (85%)	197427 (85%)	204753 (88%)	

Black	35198 (17%)	16886 (8%)	10097 (4%)	8661 (4%)	5981 (3%)	

Hispanic	34416 (17%)	22541 (10%)	20216 (9%)	18205 (8%)	13576 (6%)	

Other	5555 (3%)	5930 (3%)	5805 (2%)	6638 (3%)	7620 (3%)	

Insurance Status						<0.001

Medicare/Commercial	180433 (87%)	201681 (91%)	218819 (93%)	218483 (93%)	224437 (95%)	

Self Pay/No Insurance	5741 (3%)	4790 (2%)	4027 (2%)	4251 (2%)	4000 (2%)	

Medicaid/Other Governmental	22361 (11%)	16361 (7%)	13448 (6%)	11194 (5%)	7902 (3%)	

No. Female	114616 (55%)	120988 (54%)	126251 (53%)	123491 (53%)	120934 (51%)	<0.001

Comorbidity						<0.001

0	165450 (79%)	182736 (82%)	194925 (82%)	194940 (83%)	199529 (84%)	

1	26321 (13%)	25967 (12%)	26726 (11%)	26442 (11%)	25436 (11%)	

2	12134 (6%)	10531 (5%)	10763 (5%)	9463 (4%)	8691 (4%)	

3+	4651 (2%)	3639 (1%)	3925 (2%)	3128 (2%)	2729 (1%)	

Urban/Rural						<0.001

Non-Urban adjacent to urban (ref)	108704 (52%)	121515 (55%)	122051 (52%)	130293 (56%)	152543 (65%)	

Large Metropolitan	56499 (27%)	86496 (39%)	101347 (43%)	96531 (41%)	83842 (35%)	

Small Metropolitan	25063 (12%)	13396 (6%)	12330 (5%)	6322 (2%)	0	

Micropolitan	18291 (9%)	1466 1%)	611 (<1%)	827 (<1%)	0	

* All p-values based on Wald chi-square tests.

The likelihood of an individual's surgery being performed in a freestanding ASCs varied by their socioeconomic status (Table 3). In unadjusted analysis, patients of the lowest socioeconomic status were significantly less likely to use the ASC than patients of higher status. Adjustment for age, race, gender, insurance, comorbidity, procedural, and urbanization differences decreased the magnitude of the effect of socioeconomic status on the location of surgery. However, the lowest status group continued to be significantly less likely to use the ASC than more advantaged patient groups. Overall, the middle socioeconomic status group was the most likely group to use the ASC (OR 1.23, CI 1.21 to 1.25).

**Table 3:** Likelihood of procedure being performed in an ASC (Odds Ratio with 95% CI)

Socioeconomic Status (Quintiles)	Crude OR (95%CI)	Adjusted OR (95%CI)*
1^st ^(Lowest)	reference	reference

2^nd^	1.23 (1.22, 1.25)	1.13 (1.12, 1.15)

3^rd^	1.36 (1.35, 1.38)	1.23 (1.21, 1.25)

4^th^	1.26 (1.24, 1.27)	1.08 (1.06, 1.09)

5^th ^(Highest)	1.24 (1.22, 1.25)	1.07 (1.05, 1.09)

* Adjusted for Age, Race, Gender, Insurance Status, Type of Procedure, Comorbidity, and Level of Urbanization. Models based on n = 1,122,137 discharges

Significant interactions were found between socioeconomic status groups and race. Using the lowest quintile group as the reference, we found that for both black and white patients the likelihood of having surgery performed in an ASC increased from the lowest socioeconomic status group to the higher groups (Table 4). For Hispanic patients, the trends in ASC use were reversed. Patients from higher socioeconomic status groups were less likely to have procedures performed in ASCs compared to patients from lower groups. Using the lowest group as the reference, the OR for surgery in an ASC ranged from 0.89 (95% CI 0.83, 0.95) for the 2^nd ^quintile to 0.84 (95% CI 0.78, 0.91) for the 5^th ^quintile. For patients of other racial/ethnic groups, ASC use did not appear to vary by socioeconomic status.

**Table 4:** Racial/Ethnic Differences in Location of Surgery

Socioeconomic Status (Quintiles)	White OR (95% CI)*	Black OR (95% CI)*	Hispanic OR (95% CI)*	Other OR (95% CI)*
1^st ^(Lowest)	reference	reference	reference	reference

2^nd^	1.20 (1.13, 1.27)	1.24 (1.16, 1.33)	0.89 (0.83, 0.95)	1.04 (1.01, 1.07)

3^rd^	1.28 (1.20, 1.36)	1.28 (1.18, 1.38)	1.10 (1.02, 1.18)	1.13 (1.10, 1.16)

4^th^	1.14 (1.07, 1.21)	1.03 (0.95, 1.11)	0.88 (0.82, 0.94)	0.95 (0.92, 0.98)

5^th ^(Highest)	1.13 (1.06, 1.20)	1.17 (1.07, 1.27)	0.84 (0.78, 0.91)	0.98 (0.95, 1.00)

* reflects interaction between Socioeconomic Status and Race/Ethnicity adjusted for Age, Gender, Insurance Status, Type of Procedure, Comorbidity, and Level of Urbanization Models based on n = 1,122,137 discharges

## Discussion

Disadvantaged populations, as measured by zip code level socioeconomic status, are more likely to have outpatient surgery performed in hospitals than in ASCs. Patient race and ethnicity appear to modify the association between socioeconomic status and ASC use. For black and white patients, increased socioeconomic status continues to be significantly associated with ASC use. Conversely, Hispanic patients from lower socioeconomic status areas were more likely to use ASCs compared those residing in the higher groups. Because of the cost savings provided by ASC use for patients through lower overall costs and lower co-payments,[20] ASC use would be especially important for patients of the lowest socioeconomic status groups.

Given the improved efficiencies of ASCs over hospital outpatient departments, patients could be expected to abandon hospital based outpatient surgery. However, such a trend has not developed. Instead hospital use for outpatient surgery has remained stable, and use of ASCs has grown.[21] Barriers to the use of ASCs may exist that keep certain groups of patients in hospital outpatient departments.

One possible barrier raised by our results is patient profiling. Such profiling may be valid as in selecting patients with less comorbidity for surgery in ASCs,[22] or may be inappropriate if barriers are created for groups based on economic status, race, or ethnicity. [23-25] Since the findings of decreased ASC use in the least affluent patients were robust to control for comorbidity, sources of inappropriate profiling need to be considered.

Data from observation of physician encounters with patients supports the contention that patient profiling based on race and ethnicity may be responsible for differences in ASC use. These studies show that physicians will often recommend different procedures for the same clinical situation when the race or gender of the patient is changed.[26] In addition, economic profiling of patients by the physician may occur. Since most ASCs are for profit enterprises with significant physician ownership,[27,28] physicians have active incentives to ensure high reimbursement through these facilities. As such, similar to results seen for specialty hospitals,[29] they may discourage the use of ASCs among patients with poor insurance and lower socioeconomic status.

In addition to physician factors, structural factors in the health care system may be responsible for the utilization patterns found. As for profit enterprises,[28] investors in ASCs have financial incentives to avoid ventures where lack of reimbursement potential is perceived. These facilities may not be established in areas of lower socioeconomic status due to investor concerns about the insurance mix in the population. Thus, a physical barrier to ASC use based on community economic profiling may exist that limits the access of less advantaged patients to ASCs.

Regardless of the cause of the disparity, the findings in this study support the contention that more financially vulnerable groups are encountering a higher aggregate cost burden for their care than more advantaged groups. The benefits of ASCs in cost, convenience, and efficiency are not equitably distributed. Both physician and structural factors may be ultimately responsible for the association between socioeconomic status and ASC utilization found in this paper. Indeed, the finding of different effects of race on the likelihood of ASC use by socioeconomic status could be a result of either physician level or system level factors. Further research into the underlying reasons for these observations is needed to correct these biases in the delivery of health care.

### Study Limitations

Zip code tabulation areas (ZCTAs) were used to geocode the discharge records. Zip code level evaluations of socioeconomic status have been demonstrated to result in different parameter estimates than evaluations based on census block groups. With the larger population base in zip codes, estimates would likely be biased towards the null. Furthermore, since ZCTAs were used as the geographic unit of analysis in this study, spatial and temporal discrepancies between the zip codes reported in the SASD and the ZCTA from the census bureau exist. ZCTAs and zip codes may share the same 5 digit code while not representing the same geographic entity.[30] Usually, the spatial discontinuity between these measures of geography is small.[31,32] A potentially larger problem exists in zip code changes over time. While we used data with patient reported zip code information from 2005, the ZCTAs were last updated in 2002.[32] Thus, there is potential for mismatch between the two measures of geography. However, less than one percent of our cohort was lost due to issues of missing data, suggesting that the temporal discontinuity issue was not a significant factor in our study.

A further issue to be addressed is our inclusion of only one state, Florida, in the analysis. Florida has a more elderly population than many other states, more for profit facility ownership, no certificate of need requirements, and higher per capita health care use than other states. Despite these issues, data from Florida provided a valuable substrate for our study due to the ability to gather discharges from both the ASC and hospital environments. Furthermore, the factors that make Florida a potentially unique market, including the lack of certificate of need requirements, allow us to see ASC utilization patterns independent of regulatory forces. ASC use, and disparities in use, may be lower in states with certificate of need requirements.

Finally, patients of lower socioeconomic status often carry high burdens of comorbid illness and more severe underlying disease.[33,34] As such, they may be less appropriate candidates on average for surgery in ASCs. Although we correct for comorbidity in our analysis, subtle differences in severity of comorbid conditions cannot be addressed and may result is residual confounding. However, we believe the impact of these issues is minimized as all patients in the study had ambulatory surgery.

## Conclusion

Regardless of the cause of the disparity, patients of lower socioeconomic status likely encounter a higher cost burden for their care than people from more advantaged neighborhoods. The benefits of ASCs in cost, convenience, and efficiency are not equitably distributed. Both physician and structural factors may be ultimately responsible for the association between socioeconomic status and ASC utilization found in this paper. Indeed, the finding of different effects of race on the odds of surgery in an ASC by socioeconomic status could be a result of either physician level or system level factors. Further research into the underlying reasons for these disparities is needed to correct this inequity in health care.

## Competing interests

The authors declare that they have no competing interests.

## Authors' contributions

SS conceived of the study, performed data acquisition, provided data analysis, and drafted the paper. AS helped draft the manuscript. ZY assisted in data acquisition and data analysis. JW assisted in manuscript preparation. BH assisted in study conception, data analysis, and manuscript preparation. All authors read and approved the final manuscript.

## Pre-publication history

The pre-publication history for this paper can be accessed here:


